# Preservation of Simmental bull sperm at 0°C in Tris dilution: effect of dilution ratio and long-distance transport

**DOI:** 10.5713/ab.23.0234

**Published:** 2023-08-30

**Authors:** Shouqing Jiang, Fei Huang, Peng Niu, Jieru Wang, Xiaoxia He, Chunmei Han, Qinghua Gao

**Affiliations:** 1 College of Animal Science and Technology, Tarim University, Alar 843300, China; 2 Key Laboratory of Tarim Animal Husbandry Science and Technology, Xinjiang Production and Construction Corps, Alar 843300, China; 3 College of Life Science and Technology, Tarim University, Alar 843300, China; 4 Key Laboratory of Protection and Utilization of Biological Resources in Tarim Basin, Xinjiang Production and Construction Corps, Alar 843300, China

**Keywords:** Diluent, Long-distance Transportation, Simmental Bull

## Abstract

**Objective:**

This study aimed to assess the impact of the dilution ratio of Tris diluent, storage at 0°C, and long-distance transportation on the spermatozoa of Simmental cattle. It also validated the feasibility of the regional distribution of fresh semen.

**Methods:**

In experiment 1, semen was diluted at four dilution ratios (1:6, 1:9, 1:12, and 1:15) to determine the optimal dilution ratio of Tris diluent. In experiment 2, we assessed sperm viability, progressive motility (objectively assessed by computer-assisted sperm analyzer), and acrosome intactness in Tris dilutions kept at constant 0°C for 1, 3, 6, 9, and 12 days. We compared them to Tianshan livestock dilutions (Commercial diluent). In experiment 3, semen was diluted using Tris diluent, and sperm quality was measured before and after long-distance transport. Artificial insemination of 177 Simmental heifers compared to 156 using Tianshan Livestock dilution.

**Results:**

The outcomes demonstrated that 1:9 was the ideal Tris diluent dilution ratio. The sperm viability, Progressive Motility, and acrosome integrity of both Tris and Tianshan dilutions preserved at 0°C gradually decreased over time. sperm viability was above 50% for both dilutions on d 9, with a flat rate of decline. The decrease in acrosome integrity rate was faster for Tianshan livestock dilutions than for Tris dilutions when stored at 0°C for 1 to 6 days. There was no significant difference (p>0.05) in sperm viability between semen preserved in Tris diluent after long-distance transportation and semen preserved in resting condition. The conception rates for Tris dilution and Tianshan livestock dilution were 49.15% and 46.15% respectively, with no significant difference (p>0.05).

**Conclusion:**

This shows that Tris diluent is a good long-term protectant. It has been observed that fresh semen can be successfully preserved for long-distance transport when stored under 0°C conditions. Additionally, it is feasible to distribute semen regionally.

## INTRODUCTION

Liquid semen is currently used for bovine artificial insemination in only a few cases, and its use is limited by the season [1]. However, when only frozen semen is used, the animals have a low conception rate. This necessitates doubling the bulls required for the same breeding task, resulting in increased semen usage [2]. Preservation at 0°C is essential for efficient, economical, long-term sperm storage that reduces production costs and is easy to store. It also has essential advantages in breeding excellent animals, protecting endangered species, and international exchange of germplasm resources [3]. In China, most of the livestock are predominantly raised in remote areas, and for intensive farming models, there are many individual and cooperative farming models. Preserving semen at 0°C is more convenient for them and reduces the cost of semen preservation. The widespread use of artificial insemination in cattle is partly attributed to the availability of semen dilutions [4]. Semen in liquid nitrogen has a longer shelf life but low semen utilization. We Imagine establishing a fresh semen distribution point in each region. This is now a new model of semen distribution. The same is true for fresh milk distribution. This paper analyses the long-distance transport of semen to verify the feasibility of semen distribution.

Previous studies have shown that vibrations generated during transport can affect sperm [5]. The cost of liquid semen cannot be determined solely by the number of semen straws contained, but also by the potential need for temperature control during storage and transportation. Therefore, factors such as the type of diluent, the frequency and time of vibration, and the temperature during transportation should be considered. Additionally, a suitable quality semen diluent is crucial for extending the insemination time of liquid semen. In our research, we have developed a homemade Tris dilution, but it has not yet been thoroughly evaluated. (Grant No. ZL2007100181613, IPC Classification No.CN100581553C). Therefore, the purpose of this study was to determine the dilution ratio of semen in Tris diluent and to assess the ability of Tris diluent to preserve semen of Simmental cattle under storage at 0°C and long-distance transportation.

## MATERIALS AND METHODS

### Ethics statement

All animal handling practices are approved by the University of Tarim Animal Care and Use Committee (NO. DTU 20230120).

### Experiments

*Experiment 1*: Tris dilutions were stored at 0°C using four different dilution ratios (1:6, 1:9, 1:12, and 1:15). On the 3 days, sperm viability and Progressive Motility were observed. The ideal dilution level was determined, and subsequent tests were conducted using this ratio.

*Experiment 2*: To compare the effect of preserving semen in Tris dilutions and Tianshan livestock dilutions. Semen was collected from 6 bulls and stored at 0°C for 12 days. The viability of sperm, progressive motility (assessed using a computer-assisted sperm analyzer), and acrosome integrity were evaluated at 1, 3, 6, 9, and 12 days. The aim was to determine the impact of Tris dilution on preserving the semen of Simmental cattle at 0°C before conducting further experiments.

**Figure 1:**
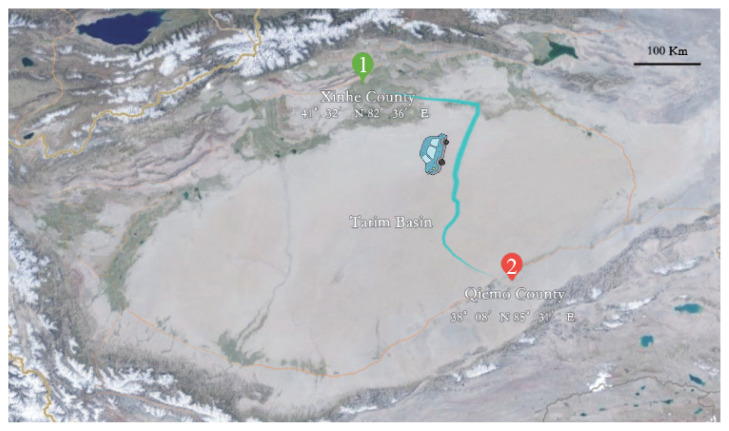
The green marker 1 is the starting point, the red marker 2 is the endpoint, and the total length is 783 km.

*Experiment 3*: To compare the effects of temperature differences, altitude differences, road conditions, and human factors on sperm quality experienced over long distances. At Dingxin Breeding Technology Co. (Xinhe County, China), we used Tris diluent to keep the semen from Simmental bulls diluted on a heated bench at 35°C. The diluted semen was cooled and equilibrated in a refrigerator at 4°C and then placed in a bubble box containing an ice-water mixture. During transport, the semen was kept in a car refrigerator at 0°C along with the foam box. The temperature was 24°C when we left Xinhe County (altitude 1,015 m, 41°32′ N 82°36′ E) in the morning, and the maximum temperature was 39°C when we passed through the Taklamakan Desert at noon. In the evening, it arrived at Qiemo County (altitude 1,295 m, 38°08′ N 85°31′ E) with a temperature of 29°C (Figure 1). To compare sperm viability, and progressive motility at the same time point, after long-distance transport versus static preservation, before performing subsequent experiments. After sperm quality testing, Simmental heifers were inseminated using semen configured with Tris dilutions that had been transported over long distances and Tianshan livestock dilutions that had been kept for the same period of time, respectively.

### Diluent preparation

Unless otherwise specified, the drugs used in this experiment were obtained from Solarbio. The proportion of citric acid in Tris diluent is reduced compared to other diluents, thus maintaining a weakly acidic environment. The inclusion of bovine serum protein mimics the natural components found in physiological conditions. It also reduces the wall-hanging of the diluent ingredients, allowing for a more homogeneous composition. The presence of vitamin C serves as an antioxidant, protecting sperm from oxidative damage caused by reactive oxygen species during the haulage process. Tris dilution basic solution formula: Tris (4.84 g), citric acid (2.68 g), bovine serum albumin (300 mg), fructose (2.0 g), adenosine triphosphate (100 mg), vitamin C (600 mg), double distilled water (136 g), penicillin (400,000 IU), streptomycin (200,000 IU). Tianshan livestock dilutions formula: Tris (6.055 g), citric acid (3.355 g), glucose (2.5 g), Egg yolk (50 mL), Pro-streptomycin (200,000 IU), double distilled water (184 g). Each 5 mL was dispensed into a vial, sealed with sealing film, and stored in a refrigerator at −80°C.

### Sperm processing

The semen used in this study was obtained from China Dingxin Seed Technology Co. Six Simmental aged 2 to 3 years were selected, and semen was collected from each bull using a sham vagina according to the protocol described by Yates et al [6]. Six bulls and 16 ejaculations of semen were collected, and the samples were evenly split into two portions. Tris dilutions were performed at four different dilution ratios (1:6, 1:9, 1:12, and 1:15). After determining the optimal dilution level of Tris diluent, semen with sperm viability greater than 70% and standard color and odor were added to two dilutions preheated at 34°C. The dilutions were then placed in a 4°C refrigerator for 2 h. Subsequently, the temperature was lowered to 0°C, and the semen was stored in a 0°C ice water mixture. The diluted semen was gently inverted and shaken every 12 h while being stored at 0°C.

### Sperm quality assessment

Sperm viability and Progressive Motility test: Sperm stored in two different dilutions at 0°C was sampled and analyzed on days 1, 3, 6, 9, and 12. Semen was mixed quickly and gently in the refrigerator. A 10 μL drop of semen was placed in a disposable counting cell and placed on a 37°C carrier table. A Sperm Class Analyzer (SCA) version 5.3 and a CPH-200 trinocular phase contrast microscope mounted on a microscope were utilized to monitor and document the motility of a minimum of 300 spermatozoa. The measurements were made three times, and the findings were averaged over the three observations.

*Sperm acrosome integrity test*: 10 μL of semen was pipetted on one side of the slide and the smooth side of the other slide used to spread the semen evenly on the slide at an angle of 35 degrees. It was air dried naturally for 5 min, then added 2 mL of formaldehyde fixative dropwise, fixed for 15 min wash, and air dried again. Grimes staining solution was added dropwise, stained for 1.5 h, and then washed with distilled water and air-dried. The prepared smears were placed under a 400× microscope to observe the number of intact spermatozoa in the acrosome.

### Simultaneous estrus and artificial insemination

In the pre-insemination period, heifers were on free range and supplemented with soy and grain diets. The Controlled Intravaginal Drug Release (CIDR, 1.56 g progesterone/stem; Yanrui Biotechnology Co., Ltd., Jiangsu, China) device was synchronized and maintained for 14 days. On 0 d, vaginal suppositories (CIDR) were placed and gonadorelin 200 μg (GnRH, 200 μg/stem; Ningbo Sangsheng Co., Ltd. Zhejiang, China) was administered intramuscularly to the heifers. Vaginal suppositories were withdrawn at 11 days, and cloprostenol sodium 0.4 mg per cow (PG, 0.2 mg/stem, Ningbo No. 2 Hormone Factory. Zhejiang, China) was injected intramuscularly. After testing the sperm quality to meet the standard of liquid semen transfusion. Two types of semen preserved in different diluents were randomly inseminated in Simmental heifers. Semen preserved in Tris diluent and transported over long distances was used to artificially inseminate 177 Simmental heifers. Fresh semen was diluted with Tianshan livestock diluent and kept for the same number of days before the insemination of 156 Simmental heifers. After the completion of artificial insemination, conception rates were tested on day 60.

### Statistical analysis

The data on semen preservation was collected, and statistical analysis was performed for each time point of preservation. All data were tested for normal distribution before analysis. All the values are presented as the mean±standard deviation. Sperm viability, cumulative viability, and acrosome integrity were analyzed by Two-way analysis of variance followed by Tukey post hoc test using SPSS 27.0 software. The chi-square test was performed for the conception rate. Statistical significance is defined when p values are less than 0.05.

## RESULTS

**Figure 2:**
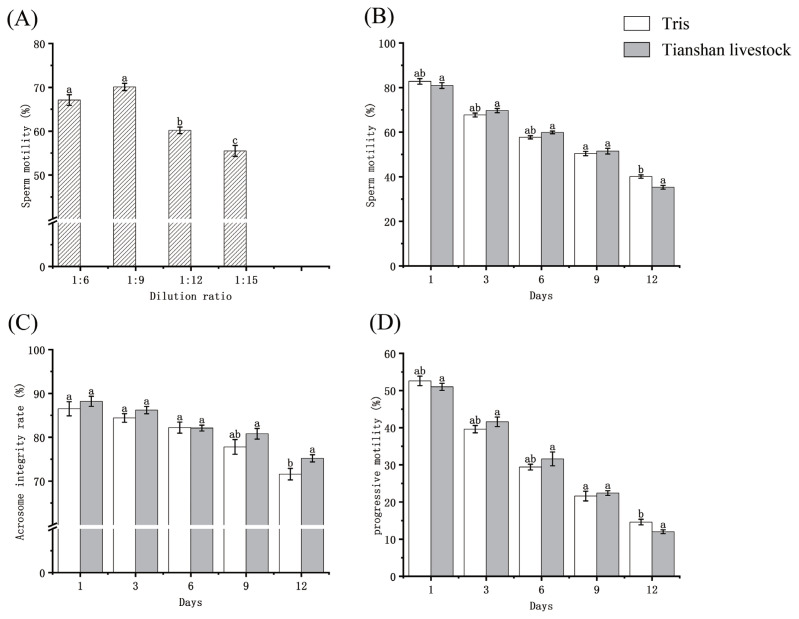
(A) Sperm viability of Tris dilutions stored at 0°C for 3 days at different dilution ratios. (B), (C), and (D) are sperm viability, acrosome integrity, and progressive motility for different days of storage at 0°C, respectively. The difference between a and b was significant at p<0.05, and the difference between a and c was significant at p<0.01.

The experimental data are presented in Figure 2 (Supplementary Table S1). Following three days of storage at 0°C in Tris dilutions, sperm viability at 1:6, 1:9, 1:12, and 1:15 ratios were 67.1±1.23, 70.1±0.84, 60.2±0.75, and 55.5±1.24, respectively (p<0.05). The sperm progressive motility was 38.5±0.76, 41.7±1.11, 35.2±0.83, and 27.9±1.18, respectively (p<0.05). The optimal dilution ratio was determined to be 1:9. On day 1, the sperm viability preserved in Tris dilution and Tianshan Livestock dilution was 82.8±1.26 and 80.9±1.29, respectively (p>0.05). Progressive motility was 52.6±1.27 and 51.0±0.96, respectively (p>0.05). Over time, sperm viability and progressive motility gradually decreased in both dilutions. There was no sharp downward trend in sperm viability and progressive motility for both dilutions in the first 9 days, and the rate of decline was flat. Until 9 days, the motility of diluted bull sperm remained above 50%.

The integrity rate of sperm acrosome gradually decreased in Tris dilution, while it was preserved in Tianshan livestock dilution. On day 1, the two dilutions preserved sperm acrosome integrity rates of 86.5±1.61 and 88.2±1.15 (p>0.05). The rate of decline of the 1 to 6 day Tianshan livestock dilutions was faster than that of the Tris dilutions. The acrosome integrity rates were 77.8±1.67 and 80.8±1.20 (p<0.05) at 9 days and 71.6±1.31 and 75.2±0.83 (p<0.05) at 12 days, respectively.

The sperm viability of Tris dilutions stored at 0°C was 85.2±1.33 before the long-distance transportation and 78.2± 1.36 after arriving in Qiemo County. At the same time, the viability of sperm preserved at rest was 80.0±0.99. There was no significant difference (p>0.05) in sperm viability after long-distance transport versus storage at 0°C in the resting state (Table 1). The semen preserved using Tris dilution was used for artificial insemination of 177 cows out of which 87 were successful, indicating a conception rate of 49.15%. Artificial insemination of 156 heads of which 72 were pregnant using ready-mixed Tianshan livestock dilution-preserved semen. The conception rate was 46.15% (Table 2), with no significant difference (p>0.05).

## DISCUSSION

The dilution multiple had a significant effect on the viability and progressive motility of spermatozoa preserved at 0°C, indicating a correlation between dilution multiple and sperm quality. Sperm viability during dilution may be negatively affected by high dilution. At dilution to a sperm concentration of 5 million, sperm viability, acrosome integrity, and plasma membrane integrity were reduced. At dilutions of 5 to 20 million sperm, there was a significant increase in motile sperm and peroxidized sperm [7]. This is consistent with the results of this experiment, which also indicates that the high dilution of semen affects the function and motility of spermatozoa.

After comparing the sperm viability at four different dilution ratios, we found that the viability of the low dilution was higher than that of the high dilution. It is important to note that too low a dilution ratio can also hurt sperm viability. This could be because sperm not only compete but also provide assistance. Individually, spermatozoa often lack the motility required to enter the oviduct [8]. However, when outside, spermatozoa can aggregate, which increases the chances of fertilization [9]. It is worth mentioning that there seems to be a connection between spermatozoa, as they tend to prefer genetically similar ones. Additionally, neighboring groups of spermatozoa can establish contact through hydrodynamic interactions, even without direct physical contact [10]. Studies have shown that when multiple groups of sperm swim together in a coordinated manner, they generate fluid movement. This phenomenon is particularly noticeable when the distance between spermatozoa is not greater than the length of a single sperm [11]. Consequently, a dilution ratio that is too large can impact the interaction between sperm and lead to an increase in energy expenditure. When semen is diluted, the absence of stimulants like albumin can lead to sperm attaching to the walls of the tubes. This behavior differs from ATP deficiency or damaged sperm [12]. We believe that this is a ‘Circumvention behavior’ of the spermatozoa. In cases where the stimulus, such as albumin, is weakened due to a high dilution, competent spermatozoa attach to the tube walls. When the environment becomes favorable and they encounter the reproductive tract from a new perspective, they are activated to perform their function. Therefore, low sperm motility should not be automatically considered a criterion for loss of viability. Sperm with low motility may still have a reasonable fertilization rate after interacting with the reproductive tract.

The sperm quality of both dilutions gradually decreases as the number of days of storage increases. Tris dilution is more effective than Tianshan livestock dilution. Zhao et al [13] used in Tris dilutions, preserved semen at 0°C, and tested semen quality at 24, 72, 144, 216, and 288 h, with similar results to the present experiment. At d 9, sperm viability was above 50%, and acrosome integrity was above 70%. The decrease in sperm quality may be attributed to the low-temperature storage of semen at 0°C. While this storage method can reduce the metabolic activity of sperm and prolong their lifespan, it can also result in sperm cold shock. This process leads to the production of H_2_O_2_ [14], which in turn decreases mitochondrial activity [15] and disrupts the mitochondrial electron transport chain [16]. Consequently, this affects sperm fatty acid peroxidation, DNA damage [17], and apoptosis. Sperm cooled in citrate exhibited reduced acrosome integrity after thawing. However, there was no significant difference observed between lactose and Tris diluent after 3 h of thawing [18]. This suggests that citrate has a suppressive effect on spermatozoa, causing a slowdown in sperm motility when frozen. The addition of citrate to Tris has been found to enhance the cleavage rate of fertilized eggs and improve the quality of embryos compared to normal diluents [19]. However, excessive citrate in diluents has been shown to reduce the sperm’s ability to penetrate cervical mucus [20]. Vitamin C, on the other hand, acts as an inhibitor of oxidative stress, increasing membrane potential difference and the rate of cleavage of fertilized eggs [21]. It plays a crucial role in maintaining sperm quality and improving embryo quality [22].

Sperm viability after long-distance transport did not significantly (p>0.05) differ from that of sperm stored at 0°C in the resting state. During the semen transportation process, we drove through various geographic environments such as cities, towns, Gobi beaches, Populus euphratica forest, and deserts. The Populus euphratica forest and the desert road are the most representative. The Populus euphratica forest is located northeast of the Taklamakan Desert, near the Tarim River basin. The road winds and twists, and sperm are susceptible to the forces in the left and right directions. The Desert Highway is in the center of the Tarim Basin, with a total length of 522 km. The desert has high temperatures and intense UV rays. In the desert road, there are numerous sections with slopes exceeding 20°. While driving, the vehicle must constantly accelerate or decelerate to gain enough climbing force and ensure vehicle stability. At this time, the sperm are susceptible to oblique upward acceleration and oblique downward deceleration forces.

Studies have shown that sperm are affected by vibrations during transport, in addition to temperature imbalances. During transportation, the vibrations caused by bumps in the car create a low-frequency vibration state, which is less intense than violent shaking. This helps slow down sperm sedimentation and prevents sperm death. The vibrations cause fluid to move along the surface of the sperm, creating bubbles that can damage mitochondria and the plasma membrane [23]. However, the semen is stored in an ice-water mixture, which counteracts some of the force by providing resistance. To minimize temperature fluctuations, the semen is stored in a closed foam box during nearly 10 h transport. For semen that has not been transported over long distances, it is to mix it once after 12 h of storage. Upon arrival in Qiemo County, no significant difference in semen quality was observed (p>0.05).

In a study conducted by Ideta et al [24], it was found that the pregnancy rate in the untreated cow group was 59.7%, significantly higher than in Tris and Tianshan livestock dilutions. This may be because of the hot climate in Qiemo County, located at the edge of the Tarim Basin. Herders raise Simmental cattle to graze near the Qarqan River, where the grass is not abundant and the forage is limited and cannot provide excellent forage for the heifers. It is worth noting that there exists a correlation between sperm viability and fertility [25]. Additionally, errors associated with artificial insemination could also contribute to the decrease in pregnancy rate. According to a report [26], it was found that a low percentage of good embryos were formed when spermatozoa had a sperm count of less than 1×10^7^/mL. The quality of embryos is affected by the quality of semen, particularly the presence of abnormal sperm heads. When it comes to achieving successful pregnancy rates, it is possible to achieve good fertilization even with semen transported over long distances, if the temperature for semen preservation is properly controlled.

Compared to frozen semen, liquid semen has a relatively short shelf life. The distribution of semen is a complex process, and it would be wasteful to degrade the quality of semen due to long-distance transportation. To establish an efficient liquid semen processing and distribution system, it is crucial to address the issues of semen availability and waste through supply and demand management. Collaboration between breeding facilities, farms, and farmers is necessary to improve this system. The breeding station should choose the right breed according to the demand and be able to provide a certain amount of semen supply.

## Figures and Tables

**Table 1:** Effects of long-distance transport on sperm quality (%)

Time	Before shipping	After shipping	static 0°C storage
Sperm motility	85.2±1.33^b^	78.2±1.36^a^	80.0±0.99^a^
Progressive motility	61.0±1.76^b^	55.6±0.24^a^	57.1±0.98^a^

a,b Values within rows with different superscripts are significantly different (p<0.05).

**Table 2:** The fertility rate of semen preserved in Tris diluent

Diluent	Inseminated (n)	Pregnancy (n)	60-d pregnancy (%)
Tris dilutions	177	87	49.15^a^
Tianshan livestock dilutions	156	72	46.15^a^

a Values within columns with different superscripts are significantly different (p<0.05).

## SUPPLEMENTARY MATERIAL

**Supplementary Table S1**. Data on the quality of sperm preservation in three groups of experiments
