# Supplementary material for: Preservation of Simmental bull sperm at 0°C in Tris dilution: effect of dilution ratio and long-distance transport
Source: Anim Biosci. 2023 Aug 30;37(2):203–9. doi: 10.5713/ab.23.0234 (PMC10766479; doi:10.5713/ab.23.0234)
Supplement: Supplementary file 1 [file ab-23-0234-Supplementary-Table-1.pdf]

Table S1: Data on the quality of sperm preservation in three groups of experiments							
Experiment 1	Dilution ratio	1：6	1：9	1：12	1：15		
Dilution Ratio	Sperm Motility (%)	66.1 ± 1.23	70.1 ± 0.84	60.2 ± 0.84	55.5 ± 1.24		
	Progressive Motility (%)	28.5 ± 0.76	31.7 ± 0.76	25.2 ± 0.83	17.9 ± 1.18		
Experiment 2	Sperm Motility (%)						
0℃ storage	Days	0	1	3	6	9	12
	Tris	91.6±1.26	82.8±1.26	67.7±0.87	57.7±0.75	50.4±0.96	40.1±0.75
	tianshan	91.3±1.39	80.9±1.29	69.7±0.94	59.9±0.63	51.5±1.27	35.3±0.84
	Progressive Motility (%)						
	Days	0	1	3	6	9	12
	Tris	50.6±1.11	49.0±1.27	29.6±0.98	19.4±0.29	11.6±1.30	4.6±0.77
	tianshan	51.6±1.26	48.6±0.26	30.6±1.28	18.6±1.87	10.4±0.44	2.0±0.31
	Acrosome integrity rate (%)						
	Days	0	1	3	6	9	12
	Tris	92.7±1.04	86.5±1.61	84.4±0.98	82.2±1.26	77.8±1.67	71.6±1.31
	tianshan	91.1±0.87	88.2±1.15	86.2±0.83	82.1±0.67	80.8±1.20	75.2±0.83
Experiment 3		Before shipping	After shipping	static 0℃ storage			
Long-distance transportation	Sperm Motility (%)	85.2±1.33	78.2±1.36	80.0±0.99			
	Progressive Motility (%)	51.0±1.76	45.6±0.24	47.1±0.98			
	conception rate						
	diluent	Inseminated (n)	Pregnancy (n)	60-d pregnancy (%)			
	Tris	177	87	49.15			
	Tianshan livestock dilutions	156	72	46.15			
